# A Machine Learning Approach to Discover Rules for Expressive Performance Actions in Jazz Guitar Music

**DOI:** 10.3389/fpsyg.2016.01965

**Published:** 2016-12-20

**Authors:** Sergio I. Giraldo, Rafael Ramirez

**Affiliations:** Music Technology Group, Machine Learning and Music Lab, Department of Communication and Technology, Pompeu Fabra UniversityBarcelona, Spain

**Keywords:** expressive music performance, jazz guitar music, ornamentation, machine learning

## Abstract

Expert musicians introduce expression in their performances by manipulating sound properties such as timing, energy, pitch, and timbre. Here, we present a data driven computational approach to induce expressive performance rule models for note duration, onset, energy, and ornamentation transformations in jazz guitar music. We extract high-level features from a set of 16 commercial audio recordings (and corresponding music scores) of jazz guitarist Grant Green in order to characterize the expression in the pieces. We apply machine learning techniques to the resulting features to learn expressive performance rule models. We (1) quantitatively evaluate the accuracy of the induced models, (2) analyse the relative importance of the considered musical features, (3) discuss some of the learnt expressive performance rules in the context of previous work, and (4) assess their generailty. The accuracies of the induced predictive models is significantly above base-line levels indicating that the audio performances and the musical features extracted contain sufficient information to automatically learn informative expressive performance patterns. Feature analysis shows that the most important musical features for predicting expressive transformations are note duration, pitch, metrical strength, phrase position, Narmour structure, and tempo and key of the piece. Similarities and differences between the induced expressive rules and the rules reported in the literature were found. Differences may be due to the fact that most previously studied performance data has consisted of classical music recordings. Finally, the rules' performer specificity/generality is assessed by applying the induced rules to performances of the same pieces performed by two other professional jazz guitar players. Results show a consistency in the ornamentation patterns between Grant Green and the other two musicians, which may be interpreted as a good indicator for generality of the ornamentation rules.

## 1. Introduction

Expressive performance actions (EPAs) such as variations in timing, dynamics, articulation, and ornamentation, are resources used by musicians when performing a musical piece in order to add expression. In classical music, EPAs are usually indicated in the score using the archetypical conventions for *articulations* (e.g., sforzando, staccato, tenuto), *ornamentation* (e.g., grace notes, trills, turns), and *tempo deviations* (e.g., ritardando, accelerando). However in jazz music, EPAs are seldom indicated in the score, and they are freely introduced in the performance by the musician based on his/her taste, background, knowledge, and playing style. Therefore, there are no concrete rules on how and when to apply them, and can not be categorized using the classical archetypical conventions.

*Expressive music performance* (EPM) research aims to understand how and in which music contexts EPAs occur in real music performances. Numerous studies in EPM have been conducted (see Palmer, 1997; Gabrielsson, 1999, 2003 for surveys) form different perspectives (e.g., psychological and cognitive). *Computational expressive music performance* studies the phenomenon using computational tools (for an overview see Goebl et al., 2008, 2014) by generating models based on data observed/measured in music performances. The resulting *computational systems for expressive music performance* (CEMP) aim to automatically generate human-like performances by introducing variations in timing, energy, and articulation obtained by computational modeling (for an overview see Kirke and Miranda, 2013).

Two main approaches have been explored in the literature to computationally model music expression. On one hand, empirical systems have been proposed, in which expressive performance rules are obtained manually from music experts. A relevant example of such approach is the work of the KTH group (Bresin and Friberg, 2000; Friberg, 2006; Friberg et al., 2006). Their *Director Musices* system incorporates rules for tempo, dynamic, and articulation transformations. Other examples include the Hierarchical Parabola Model by Todd (1989, 1992, 1995), and the work by Johnson (1991). Johnson developed a rule-based expert system to determine expressive tempo and articulation for Bach's fugues from the Well-Tempered Clavier. The rules were obtained from two expert performers. Livingstone et al. (2010) report on a rule based system for emotion modeling of score and performance in which rule generation parameters were generated using *analysis-by-synthesis*. On the other hand, learning systems obtain expressive performance models by applying machine learning techniques to the data extracted from music performance recordings. For example, neural networks have been applied by Bresin (1998) to model piano performances, and by Camurri et al. (2000) to model nine different emotions (mapped on a 2-D space) in flute performances. Rule-based learning algorithms together with clustering algorithms have been applied by Widmer (2003) to discover general piano performance rules. Other piano expressive performance systems worth mentioning are the ESP piano system by Grindlay (2005) in which Hidden Markov Models were applied to generate expressive performances of piano music consisting of melody and chord progressions, and the generative performance system of Miranda et al. (2010) in which genetic algorithms are used to construct tempo and dynamic curves.

Most of the expressive performance systems proposed target classical piano music. Exceptions include the expressive jazz saxophone modeling approaches of Arcos et al. (1998) who use case-based reasoning, and Ramírez and Hazan (2006) who use inductive logic programming. Maestre et al. (2009) combine machine learning techniques and concatenative synthesis to synthesize jazz saxophone expressive performances. Most of these systems consider performances with simple ornaments, i.e., one-note ornamentations (e.g., grace notes or one passing notes). In previous work (Giraldo, 2012; Giraldo and Ramírez, 2015a,b,c, 2016), we applied machine learning techniques to model expressive performance actions in jazz guitar performances, which include complex ornaments. However, little attention was paid to the perspicuity of the extracted models in terms of its musical interpretation.

In this paper, we induce expressive performance rules by applying machine learning methods. Concretely, we apply a propositional rule learner algorithm to obtain expressive performance rules from the data extracted from commercial audio jazz recordings and its respectives scores. We are interested in rules characterizing EPAs, i.e., variations in timing (*onset* and *duration* deviation), *energy* (loudness), and *ornamentation* (i.e., insertion and deletion of an arbitrary number of melody notes) in jazz guitar music. To achieve this, we extract score descriptors from the scores and calculate EPAs from the resulting alignment deviations between the scores and its corresponding audio performances. Later, we apply feature selection and machine learning algorithms to induce rule models for the considered EPAs (onset, duration, energy, and ornamentation). Finally, we evaluate the accuracy of each of the models obtained, discuss the similarities between the expressive induced rules and the ones reported in the literature, and asses the generality of the models by comparing the actions predicted by the induced rules to performances by two other professional guitar players.

## 2. Materials and methods

### 2.1. Materials

The music material considered in this work is presented in Table 1, and consists of 16 commercial recordings of Grant Green, and their corresponding commercially available music scores obtained from (The real book, 2004), a compilation of jazz pieces in the form of *lead sheets*. The collected music scores contain melodic and harmonic information, i.e., main melody and chord progressions. The instrumentation for most of the pieces consists of guitar (g), piano (p), double bass (b), and drums (d). Details can be found in Table 1.

**Table 1:** **Recordings list containing album, recording year, instrumentation (g, guitar; p, piano; b, double bass; and d, drums), piece name, and performer(s)**.

**Album**	**Recording year**	**Personel/Instrumentation**	**Name**	**Author**
Standard	1961	G. Green (g)	All the things you are	J. Kern
		W. Ware (b)	If I had you	Cambell and Connelly
		A. Harewood (d)	I'll remember April	G. de Paul
			I Remember you	V. Schertzinger
			Love walked in	G. Gershwin
Goodens Corner	1961	G. Green (g)	On green dolphin street	B. Kaper
		S. Clark (p)	What is this thing called love	C. Porter
		S. Jones (b)		
		L. Hayes (d)		
Nigeria	1962	G. Green (g)	Airegin	S. Rollins
		S. Clark (p)		
		S. Jones (b)		
		A. Blakey (d)		
Green Street	1962	G. Green (g)	Alone together	A. Schwartz
		B. Tucker (b)	Moon river	H. Mancini
		D. Bailey (d)	Round about midnight	T. Monk
Born to be blue	1962	G. Green (g)	If I should lose you	R. Rainger
		S. Clark (p)	My one and only love	G. Wood
		S. Jones (b)		
		L. Hayes (d)		
Oleo	1962	G. Green (g)	Tune up	M. Davies
		S. Clark (p)		
		S. Jones (b)		
		L. Hayes (d)		
Matador	1964	G. Green (g)	My favorite things	Rogers and Hammerstein
		McC. Tyner (p)		
		B. Cranshaw (b)		
		E. Jones (d)		
I want to hold your hand	1965	G. Green (g)	Speak low	K. Weill
		L. Young (o, b)		
		E. Jones (d)		

### 2.2. Methods

**Figure 1:**
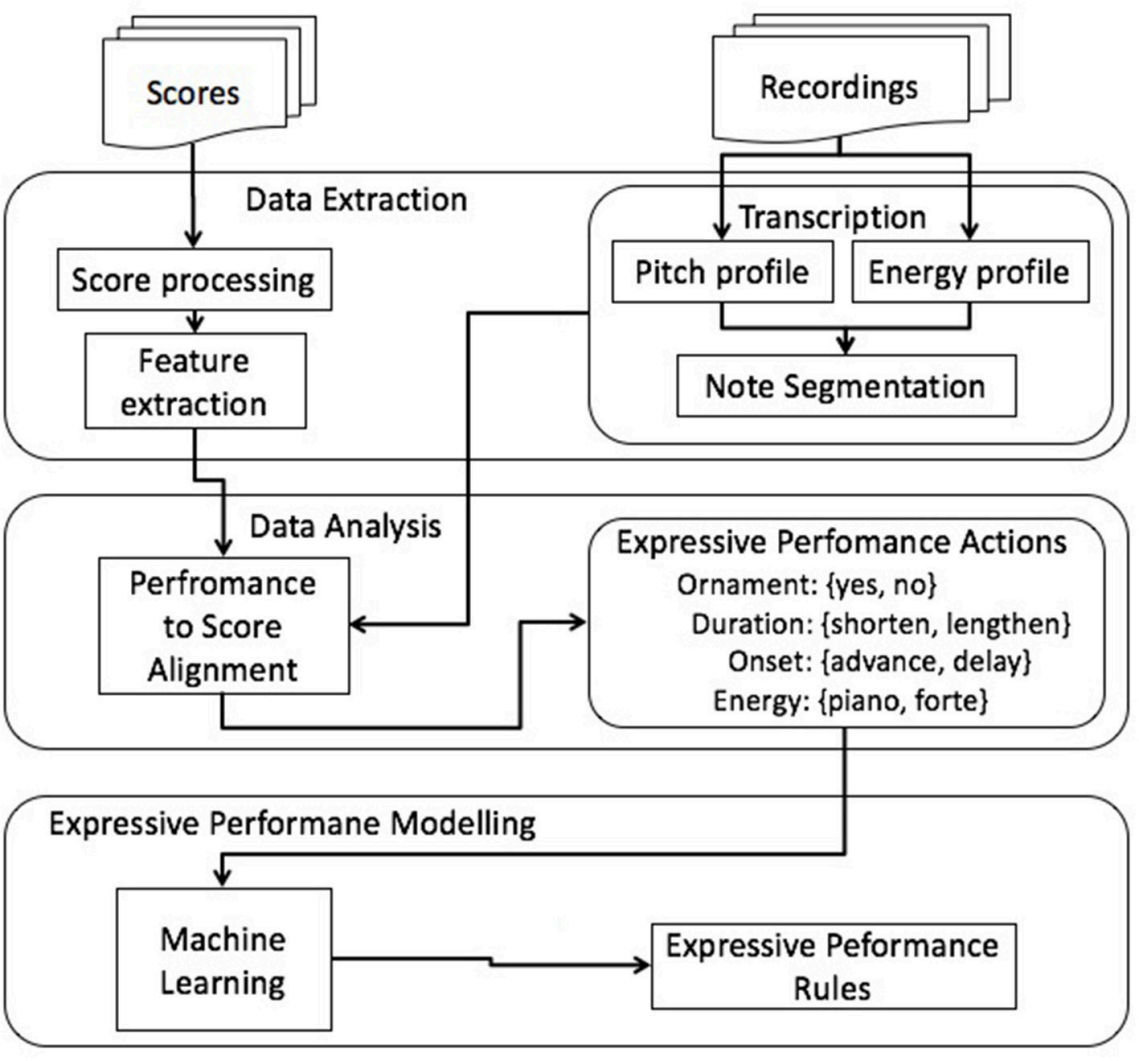
**General framework for EPAs modeling**.

The general research framework of this investigation (depicted in Figure 1) is based on our previous approach to jazz guitar ornament prediction (Giraldo, 2012; Giraldo and Ramírez, 2015a,b,c). It consists of three main blocks: data extraction, data analysis, and expressive performance modeling.

#### 2.2.1. Data extraction

In the data analysis block, both the scores and the recordings are gathered and parsed to obtain a machine readable representation. Data analysis consists of three main parts: score processing, feature extraction, and recordings transcription.

##### 2.2.1.1. Score processing

Each score was re-written using an open source software for music notation (Froment et al., 2011), and then converted to MusicXML format containing note onset, duration and tempo information, as well as contextual information (e.g., key, chords, mode). In each piece, *tempo* and *key* were adapted to match the recordings. Ambiguity in chord information in the scores was resolved as shown in **Table 4** (Notice that the chords shown in the table are listed so that they fall within an octave). Each section of the piece's melody was recorded once (i.e., no repetitions nor solos were recorded), e.g., for a piece with a (typical) *AABA* musical structure, only the sections *A* and *B* were considered.

##### 2.2.1.2. Feature extraction

**Figure 2:**
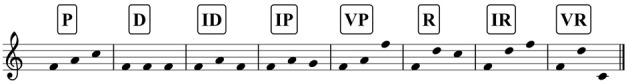
**Basic Narmour structures P, D, R, and ID, and their derivatives VR, IR, VP, and IP**.

Score notes were characterized by automatically extracting descriptors for each note, (Giraldo, 2012; Giraldo and Ramírez, 2015a,b,c). We implemented our own feature extraction library for computing all the reported features, with the exception of the *perceptual features* for which we used the methods provided by the *miditoolbox* (Eerola and Toiviainen, 2004). The complete list of extracted featuresare summarized in Table 2. Descriptors were categorized into four categories, as follows:

- **Nominal descriptors** refer to intrinsic properties of the notes (e.g., pitch, duration). *Duration* and *onset* were measured in seconds and beats, as pieces were recorded at different tempos. Tempo changes within a piece (e.g., ritardando, doubled tempo sections) were taken in consideration when performing beat-tracking (see Section 2.2.1.3). *Onset in bar* refers to the beat within a measure, and its maximum value (*bpb*) refers to the beats per bar (e.g., four in a 4/4 time signature).
- **Neighbor descriptors** refer to the note's immediate musical context given by the properties of neighboring notes (e.g., interval with previous and next note, pitch of previous and next note). Previous and next *inter-onset distance* is the distance between the onset of two consecutive notes.
- **Contextual descriptors** refer to properties of the piece in which the note appears (e.g., mode, key, chord). The *Key* descriptor refers to the piece key, and was encoded using the *circle of fifths* (e.g., *Bb* = −1, *C* = 0, *F* = 1). For some calculations (e.g., *note to key* in Table 2) a linear representation of the notes (e.g., *C* = 0, *C#*/*Db* = 1, *D* = 2) was used instead. Melodic analysis is captured with the *note to key* and *note to chord* interval descriptors. They specify the interval of each note with respect to the key and to the concurrent chord's root, respectively. *Is a chord note* is a boolean descriptor that indicates if the current note belongs to the notes comprising the ongoing chord, according to **Table 4**. *Metrical strength* categorize notes occurring at strong or weak beats within a bar, according to the time signature of the piece, as shown in Table 3. The *Phrase* descriptor was computed using the melodic segmentation approach by Cambouropoulos (1997), which indicates the probability of each note being at a phrase boundary. Probability values were used to decide if the note was a *boundary note*, annotated as either *initial (i)* or *ending (e)*. Non-boundary notes were annotated as *middle (m)*.
- **Perceptual descriptors** are inspired by music perception and cognition models. Narmour's implication-realization model (Narmour, 1992) proposes eight basic melodic structures based intervallic expectation in melodies. The basic Narmour structures (P, D, R, and ID) and their derivatives (VR, IR, VP, and IP) are represented in Figure 2. Symbols refer to prospective or retrospective (shown in parenthesis in the Range column of Table 2) realization. Schellenberg (1997) simplified and quantified Narmour's model into five principles: *registral direction, intervallic difference, registral return, proximity*, and *closure*. *Tonal stability* (Krumhansl and Kessler, 1982) represents the degree of belonging to the (local) key context. *Melodic attraction* (Lerdahl, 1996) measures the *weight* (*anchoring strength*) of the pitches across the pitch space. *Tessitura* and *mobility* are measures proposed by Von Hippel (2000). *Tessitura* is the standard deviation of the pitch height distribution and predicts the listener expectation of the tones being close to the median pitch. *Mobility* is based on the intuition that a melody is constrained to its tessitura and therefore melodies change direction after long intervals otherwise they will fall outside their comfortable range. This measure is calculated using *one lag* autocorrelation between consecutive pitches.

**Table 2:** **Note Description**.

**Descriptor**		**Units**	**Range**	**Discrete labels**
Nominal	Duration	s	[0, +∞]	{*verylarge, large, nominal, short, veryshort*}
	Duration	beats	[0, +∞]	–
	Onset	s	[0, +∞]	–
	Onset	beats	[0, +∞]	–
	Onset in Bar	beats	[0, +*bpb*]	–
	Pitch	semitones	[1, 127]	–
	Chroma	semitones	[0, 11]	{*C, C#*/*Db, D, D#*/*Eb, E*,
				*F, F#*/*Gb, G, G#*/*Ab, A, A#*/*Bb, B*}
Neighbor	Prev. duration	s	[0, +∞]	{*verylarge, large, nominal, short, veryshort*}
	Prev. duration	beats	[0, +∞]	–
	Next duration	s	[0, +∞]	{*verylarge, large, nominal, short, veryshort*}
	Next duration	beats	[0, +∞]	–
	Prev. interval dir	semitones	[−60, 60]	{*ascending, unison, descending*}
	Prev. interval	semitones	[−60, 60]	{*large, small*}
	Next interval dir	semitones	[−60, 60]	{*ascending, unison, descending*}
	Next interval	semitones	[−60, 60]	{*large, small*}
	Prev. inter-onset dist.	s	[0, +∞]	{*verylarge, large, nominal, short, veryshort*}
	Next. inter-onset dist.	s	[0, +∞]	{*verylarge, large, nominal, short, veryshort*}
Context	Measure	bars	[0, +∞]	–
	Tempo	bpm	[30, 260]	{*Up*−*tempo, medium, moderate, slow*}
	Key	semitones	[0, 11]	{*C, C#*/*Db, D, D#*/*Eb, E*,
				*F, F#*/*Gb, G, G#*/*Ab, A, A#*/*Bb, B*}
	Mode	label	–	{*major, minor*}
	Note to Key	semitones	[0, 11]	–
	Chord root	semitones	[0, 11]	–
			–	{*+, 6, 7, 7#11, 7#5, 7#9, 7alt*
	Chord type	label	–	7*b*5.7*b*9, *Maj*7, *dim, dim*7,
			–	*m, m*6, *m*7, *m*7*b*5, *major*}
	Chord func.		–	{*dom, maj, min, dim, aug, hdim, NC*}
	Note to chord	semitones	[0, 11]	–
	Is chord note	boolean	–	{*true, false*}
	Metrical Strength	label	–	{*Verystrong, Strong*,
				*Weak, Veryweak*}
	Phrase	label	–	{*initial, middle, final*}
Perceptual	Narmour I-R struc.	label	–	{*P, D, R, ID*, (*P*), (*D*), (*R*),
			–	(*ID*), *VR, IR, VP, IP*, (*VR*),
			–	(*IR*), (*VP*), (*IP*), *dyadic, monadic*}
	Nar. Reg. Dir.	boolean	{0, 1}	–
	Nar. Inter. Diff.	boolean	{0, 1}	–
	Nar. Reg. Ret.	int	{0, 1, 2, 3}	–
	Nar. Proximity	int	{0, 1, 2, 3, 4, 5, 6}	–
	Nar. Closure	int	{0, 1, 2}	–
	Consonance	int	{0, 10}	–
	Tonal stability	int	{0, 10}	–
	Melodic Attraction	%	{0, 1}	–
	Tessitura	semitones	[0, +∞]	–
	Mobility	%	{0, 1}	–

**Table 3:** **Strength at beat occurrence, for different time signatures**.

**Time signature**	**Very strong**	**Strong**	**Weak**	**Very weak**
4/4	Beat 1	Beat 3	Beats 2 and 4	Other
3/4	Beat 1	None	Beats 2 and 3	Other
6/8	Beat 1	Beat 4	Beats 2, 3, and 6	Other

Because our aim is to obtain interpretable rules from a musical perspective, a set of numerical descriptors were discretized into categorical features, according to the fourth column of Table 2. For example, *duration in seconds* was discretized into classes *very large, large, nominal, short*, and *very short*. We defined duration thresholds in seconds according to the data distribution over the quantization bins, as follows:

(1) $$duration_{nom}(n)=\{\begin{array}{ll} verylarge & \text{ if }ds_{n}\geq 1.6s.\\ large & \text{ if }1.6s.\leq ds_{n}<1s.\\ nominal & \text{ if }1s.\leq ds_{n}<0.25s.\\ short & \text{ if }0.25s.\leq ds_{n}<0.125s.\\ veryshort & \text{ if }ds_{n}\leq 0.125s. \end{array}$$

Interval sizes were categorized into *small* and *large* based on the Implication-Realization model of Narmour (Narmour, 1992), which assumes that intervals smaller/larger than 6 semitones are perceived to be small/large.

Tempo indications in jazz often are refereed based on the performance style (e.g., Bebop, Swing) or on the sub-genre of the piece (e.g., medium, medium up swing, up tempo swing). However, ambiguity on the BPM range for which this categorization corresponds exists among performers. In this section the discretization of the tempo of the piece was performed based on the performers' preferred tempo clusters found by Collier and Collier (1994). In the study, the tempo of several jazz recordings datasets are analyzed and preferred tempo clusters of performers are found at 92, 117, 160, and 220 bpm. The study is based on the assumption that tempos in the range of 4 tempo cluster (attractor) may gravitate toward it. Based on this, we defined four different bpm ranges around each cluster and labeled it as follows.

(2) $$tempo_{nom}(n)=\{\begin{array}{ll} Up-tempo & \text{ if }t_{n}\geq 180\\ Medium & \text{ if }180>t_{n}\geq 139\\ Moderate & \text{ if }139>t_{n}\geq 105\\ Slow & \text{ if }105>t_{n} \end{array}$$

Chord function was calculated based on the chord simplification rules by Hedges et al. (2014), in which the notation of the chord type (e.g., *Ebmaj*7) is simplified according to the harmonic function of the chords. In this study we adapted the rules according to make them consistent according to the chord degree definitions given in Table 4, as follows:

(3) $$chord_{func}(n)=\{\begin{array}{ll} dom & \text{ if}[4,10]\in chord\;degrees\\ maj & \text{ if}[4]\in chord\;degrees\wedge [10]\notin \\ \; & \;chord\;degrees\\ min & \text{ if}[3,7]\in chord\;degrees\\ dim & \text{ if}([0,3,6,]\vee [0,3,6,9])\\ \; & \;=chord\;degrees\\ aug & \text{ if}[\#5,+]\subset cht_{n}\\ hdim & \text{ if}[0,3,6,10]=chord\;degrees\\ dom & \text{ if}[10]\in chord\;degrees\wedge [sus]\subset cht_{n}\\ maj & \text{ if}[10]\notin chord\;degrees\wedge [sus]\subset cht_{n}\\ NC & \text{ if}nochord \end{array}$$

**Table 4:** **Chord description list**.

**Chord type**	**Intervals**
major	0 4 7
m (minor)	0 3 7
sus2	0 2 7
sus4	0 5 7
Maj7	0 4 7 11
6th	0 4 7 9
m7	0 3 7 10
m6	0 3 7 9
mMaj7	0 3 7 11
m7b5	0 3 6 10
dim	0 3 6 9
7th	0 4 7 10
7#5	0 4 8 10
7b5	0 4 6 10
7sus	0 5 7 10
Maj9	0 2 4 7 11
6/9	0 2 4 7 9
m9	0 2 3 7 9
9th	0 2 4 7 10
7b9	0 1 4 7 10
7#9	0 3 4 7 10
13	0 2 4 7 9 10
7b9b13	0 1 4 7 8 10
7alt	0 1 3 4 6 8 10

##### 2.2.1.3. Recordings transcription

In order to extract the predominant melody pitch profile from the recordings audio mix (containing guitar, double bass, drums, and piano), we applied an optimized version of the *Melodia* algorithm (Salamon and Gómez, 2012). We optimized the algorithm parameters related to spectral peak distribution thresholds, and time and pitch continuity thresholds to best detect the guitar melody in the audio mix. This optimization was implemented using *genetic algorithms* (Giraldo and Ramírez, 2014). An energy profile of the melody was obtained by manipulating the *Melodia* algorithm and forcing it to output its *confidence value* frame by frame instead of the detected *pitch profile segment* mean. From the pitch profile of the guitar, we calculated a MIDI representation of the melody by segmenting it into notes (Mcnab et al., 1996; Bantula et al., 2014; Mauch et al., 2015). Note onsets and offsets were obtained based on pitch changes and energy adaptative thresholds. Transcription errors were removed using heuristic rules based on minimum note/gap duration, defined according to human perception thresholds (Woodrow, 1951).

#### 2.2.2. Data analysis

##### 2.2.2.1. Score to performance alignment

**Figure 3:**
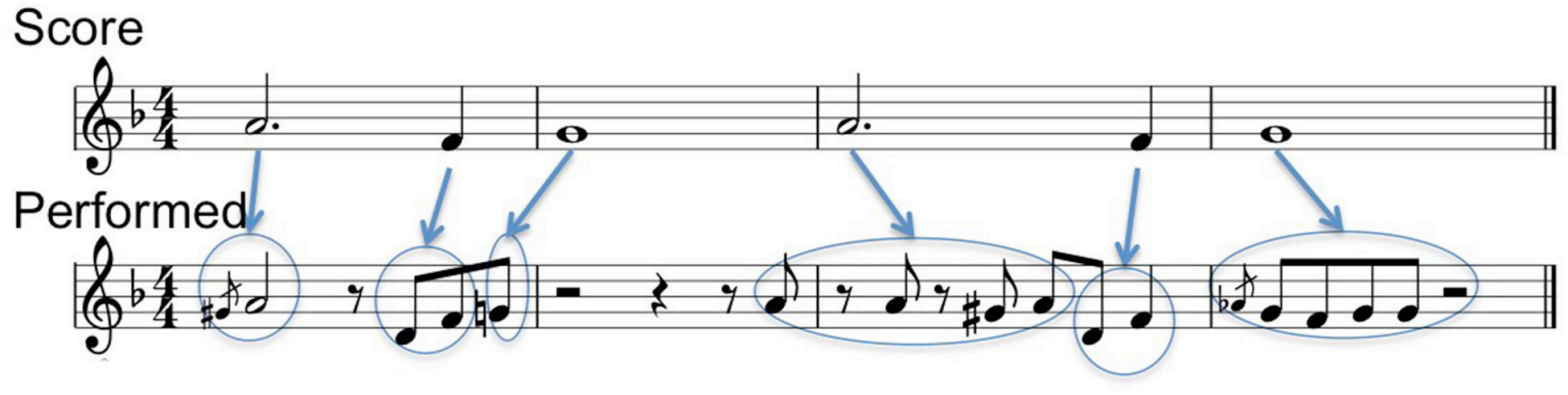
**Parent score notes (top) to performance notes (bottom) alignment example**.

Melodic ornaments in jazz consist not only of the archetypical classical music ornaments (e.g., trills, appogiaturas) but also of sets of small phrases, which are part of the jazz idiom and are used by performers based on their musical background and/or knowledge. In this context, score to performance alignment is a very challenging task as there are no clear rules about which notes on the performance correspond to which notes in the score (see Figure 3). The ornamentation alignment problem is addressed by Grachten et al. (2006) using edit-distance. Following a similar approach, we addressed this problem by applying *Dynamic Time Warping* techniques to match performance and score note sequences (Giraldo and Ramírez, 2015d). Our system automatically aligns performance notes to score notes using a distance cost function based on onset, pitch, and duration deviations, as well as deviations based on short ornament-phrase-onset/offset level. These deviations over ornament-phrase-onset/offset are calculated based on the assumption that the notes conforming the ornament are played legato, forcing the algorithm to map a score parent note to the complete set of child notes conforming the ornament in the performance sequence. After the calculation of a similarity matrix of the note events of the score against the performance, an optimal path is found in which vertical paths corresponds ornamented notes and diagonal paths corresponds one to one note correspondence (i.e., not ornamented notes). A detailed description of our aligning method can be found in Giraldo and Ramírez (2016).

##### 2.2.2.2. Expressive performance actions calculation

**Figure 4:**
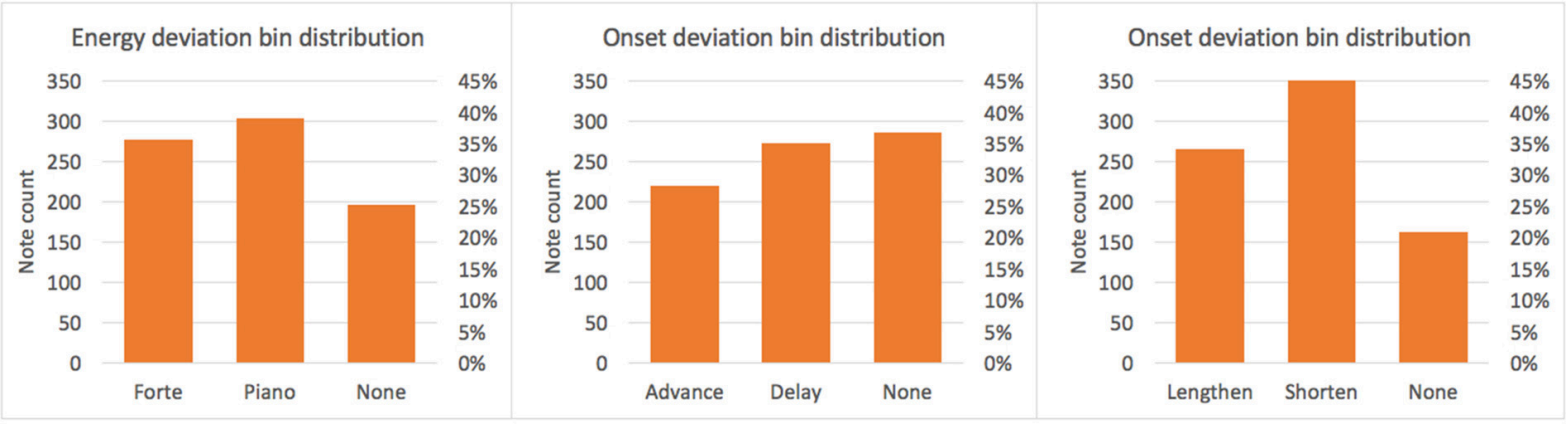
**Distribution over quantized bins of performance actions classes**.

Score notes aligned to exactly one performance note were labeled as *non-ornamented*, whereas score notes aligned to several performance notes (as well as omitted ones) were labeled as *ornamented*. Performance action deviations in duration, onset, and energy were discretized into classes as shown in Table 5. Duration was discretized into *lengthen, shorten*, and *none*; onset into *advance, delay*, and *none*; and energy into *piano, forte*, and *none*. A note is considered to belong to class *lengthen/shorten*, if its performed duration one *semiquaver* longer/shorter (or more/less) than its duration according to the score. Otherwise, it belongs to class *none*. Classes *advance, delay*, and *none* are defined analogously. A note is considered to be in class *forte/piano* if it is played louder/softer than the mean energy of the piece plus/minus 20% and in class *none* otherwise. The quantization boundaries were selected empirically by considering thresholds which seemed reasonable form a musical perspective, that at the same time produce relatively balanced distributions (see Figure 4). Finally, each pair of aligned score and performance *parent* notes were annotated along with the score note description, and the corresponding measured EPA on a database.

**Table 5:** **Expressive performance actions**.

**PA**	**Classes**
Ornamentation	{*yes, no*}
Durartion ratio	{*shorten, lengthen, none*}
Onset deviation	{*advance, delay, none*}
Energy ratio	{*piano, forte, none*}

#### 2.2.3. Expressive performance modeling

##### 2.2.3.1. Learning task

We explored machine learning techniques to induce models for predicting the different expressive performance actions defined above. Concretely, our objective is to induce four classification models M1, M2, M3, and M4 for ornamentation, note duration, note onset, and note energy, respectively. The models are of the following form:

M1(FeatureSet)→Ornamentation

M2(FeatureSet)→Duration

M3(FeatureSet)→Onset

M4(FeatureSet)→Energy

Where *M*1, *M*2, *M*3, and *M*4 are functions which take as input the set of features (*FeatureSet*) shown in Table 2, and *Ornamentation, Duration, Onset*, and *Energy* are the set of classes defined above for the corresponding performance actions.

##### 2.2.3.2. Learning algorithm

We applied Ripper (Cohen, 1995), a rule learner algorithm. This algorithm is an optimized version of the sequential covering technique used to generate rules (e.g., PRISM algorithm by Cendrowska, 1987). The main motivation for applying the Ripper algorithm was that Ripper examines the classes in ascending order, starting with the minority class, which is very convenient in our problem set, as the classes for ornamentation are unbalanced. i.e., the percentage of ornamented notes is considerably lower than the percentage of non-ornamented ones. Thus, the covering algorithm approach will try to isolate first the minority class (i.e., the class of *ornamented* notes).

Ripper evaluates the quality of rules using heuristic measures based on coverage (i.e., how much data they cover) and accuracy (i.e., how many mistakes they make). Once a rule is obtained the instances covered by the rule are removed from the data set, and the process iterates to generate a new rule, until no more instances are left. We used the WEKA library implementation of RIPPER (Hall et al., 2009).

##### 2.2.3.3. Feature selection

Automatic feature selection is a computational technique for identifying the most relevant features for a particular predictions task. Our aim is to identify the features which contain the most significant information for predicting the different expressive performance actions studied. We considered the *Wrapper* feature selection method, in which the selection is performed based on the accuracy obtained over different feature subsets for predicting the EPA (wrapper feature selection). The most relevant feature subsets for each performance action are shown in Table 6.

**Table 6:** **Most relevant features for each performance action obtained by both filter and wrapper feature selection**.

**EPA**	**Selected features**
Ornament	Duration (s)
	Next duration (beats)
	Phrase
	Next Interval
	Next duration (s)
Duration	Duration (s)
	Narmour
	Duration (beats)
	Met. Strength
	Phrase
Onset	Tempo
	Duration (s)
	Next duration (s)
	Prev. duration (s)
	Chord Type
Energy	Pitch
	Tempo
	Narmour
	Key
	Metrical strength

## 3. Results

### 3.1. Expressive performance rules

The expressive performance models induced consist of sets of conjunctive propositional rules which define a classifier for the performance actions, i.e., ornamentation, and duration, onset, and energy deviation. These rules capture general patterns for classifying the musician's expressive decisions during performance.

The set of induced expressive performance rules for each performance action is shown bellow. A rule is expressed as

IF (condition) THEN (action)

where action computes a deviation of an specific EPA.

#### 3.1.1. Ornamentation rules

- O1: *IF duration of note is very long THEN ornament note*
- O2: *IF duration of note is long AND note is the final note in a phrase THEN ornament note*
- O3: *IF duration of note is long AND next note's duration is long THEN ornament note*
- O4: *IF note is the 3rd note in an IP (Narmour) structure AND previous note's duration is not short AND next note's duration is short THEN ornament note*.

The first ornamentation rule (i.e., *IF duration of note is very long THEN ornament note*) specifies that if a note's duration is very long (i.e., longer than 1.6 s) then it is predicted as ornamented with a precision of 0.79 (calculated as the proportion of *true positives* over the sum of *true positives* plus *false positives*). The precondition of this rule is fulfilled by 111 notes in the data set from which 88 are actually ornamented and 23 are not. This rule makes musical sense since long notes are likely to be ornamented. The second ornamentation rule (Rule O2) is similar in spirit, it specifies that if a note's duration is long (i.e., longer than 1 s) and this note is the ending note of a musical phrase, then it is predicted as ornamented with a precision of 0.74. Thus, this rule relaxes the constraint on the duration of the note but requires that the note appears at the end of a phrase in order to classify it as ornamented. The rule captures the intuition that phrase boundary notes (in this case notes at the ending of a phrase) are more likely to be ornamented. Rule O3 and Rule O4 add conditions about the duration of neighboring notes (i.e., next and previous notes) in order to classify notes as ornamented. The intuition of these rules is that notes may be ornamented by using part of the duration of the neighboring notes.

#### 3.1.2. Duration rules

- D1: *IF note is the final note of a phrase AND the note appears in the third position of an IP (Narmour) structure THEN shorten note*
- D2: *IF note duration is longer than a dotted half note AND tempo is Medium (90–160 BPM) THEN shorten note*
- D3: *IF note duration is less than an eighth note AND note is in a very strong metrical position THEN lengthen note*.

#### 3.1.3. Onset deviation rules

- T1: *IF the note duration is short AND piece is up-tempo (*≥ *180 BPM) THEN advance note*
- T2: *IF the duration of the previous note is nominal AND the note's metrical strength is very strong THEN advance note*
- T3: *IF the duration of the previous note is short AND piece is up-tempo (*≥ *180 BPM) THEN advance note*
- T4: *IF the tempo is medium (90–160 BPM) AND the note is played within a tonic chord AND the next note's duration is not short nor long THEN delay note*

#### 3.1.4. Energy deviation rules

- E1: *IF the interval with next note is ascending AND the note pitch not high (lower than B3) THEN play piano*
- E2: *IF the interval with next note is descending AND the note pitch is very high (higher than C5) THEN play forte*
- E3: *IF the note is an eight note AND note is the initial note of a phrase THEN play forte*.

The rules about duration and onset transformations involve conditions that refer to note duration, metrical strength, and tempo. Long notes in medium tempo pieces are likely to be shortened (Rule D2), while short notes appearing in strong metrical positions are lengthened (Rule D3). The first onset rule (Rule T1) states that short notes in up-tempo pieces likely to be advanced, while Rule T2 constrains the first rule stating to advance notes that occur within a sequence of short notes. On the other hand, a note is delayed if it belongs to a medium tempo (i.e., 90–160 BPM) piece and it is played within a tonic chord and succeeded by a medium length note (Rule T4). Finally, energy deviation rules contain conditions that refers to the direction of the interval with respect to the next note. Rule E1 states that notes occurring in a low pitch register and in an ascending interval are played softer, whereas notes coming from higher pitch registers and in a descending intervals are played forte (Rule E2). Rule E3 states that a note occurring at the beginning of a phrase is accentuated by playing it forte.

## 4. Discussion

### 4.1. Feature selection analysis

As can be seen from the feature selection analysis (Table 6), the most influential descriptors for predicting ornamentation in the investigated performance recordings are *duration in beats* and *Duration in seconds*. This may be explained by the fact that it is easier and more natural to ornament longer notes as opposed to shorter ones. In addition to allowing more time to plan the particular ornamentation when playing long notes, it is technically simpler to replace a long note with a sequence of notes than it is for shorter notes. *Duration in seconds* represents the absolute duration of a note, while *duration in beats* represents the relative duration of a note measured in beats. In general, notes with same *duration in beats* values may vary considerably depending on the tempo of the piece to which they belong. Intuitively, it is the duration of a note in seconds which is the most important feature according to what we have discussed above, so the fact that one feature selection method (e.g., filter feature selection) ranked first the duration in beats feature may indicate that the variation in tempo in the pieces in our data-set is not too important to show this fact. Similarly, *next duration in beats* and *next duration in seconds* have been found to be very informative features by the feature selection algorithms. This may be explained as in the case of the *duration in beats* and *duration in seconds* features: notes that are followed by long notes are more likely to be ornamented since it is possible to introduce extra notes by using part of the duration of the following note.

*Next interval* and *NarNext interval* are other informative features for ornamentation prediction as detected by the feature selection algorithms. The importance of *Next interval* may be interpreted by the fact that notes that are followed by notes forming an interval of more than 1 or 2 semitones may be ornamented by inserting one or more approximation notes. *Phrase* has been also identified as informative. This confirms our intuition that notes in phrase boundaries are more likely to be ornamented. *Nar* is related to the degree of expectation of a note's pitch, so the fact that this feature is among the five most informative features for predicting ornamentation may be due that musicians tend ornament highly expected notes in order to add variation and surprise to the performed a melody. This is interesting because according to Narmour's theory these expectations are innate in humans so it may be the case that the choice to ornament expected/unexpected notes can be the results of an intuitive and unconscious process.

As expected, the most informative features for predicting ornamentation include both temporal (e.g., *Duration in seconds* and *Duration in beats*) and melodic features (e.g., *Next interval* and *Nar*). They involve not only properties of the note considered, but also properties that refer to its musical context, i.e., its neighboring notes (e.g., *Next duration, Next interval, Phrase*, and *Nar*). Similar results were obtained for the other expressive performance actions (i.e., duration, onset, and energy variations): Temporal features of the note considered and its context (e.g., *Duration in seconds, Duration in beats, Next duration*, and *Prev duration*) are found to be informative, as well as melodic features (e.g., *Pitch, Next interval*, and *Nar*). Interestingly, *Pitch* was found to be the most informative feature for energy prediction. This may be explained by the tendency of the performer to play higher pitch notes softer than lower pitch ones. It could be argued that this finding might be an artifact of the loudness measure in combination with the instrument acoustics, i.e., a higher pitched note, even if it is played by the musician with the same intensity, produces less sound. However, we discarded this possibility for two main reasons: Firstly, a high quality electric guitar should produce an even level of loudness in all its tesitura (i.e., across the fretboard). Secondly, a professional player would adjust the force applied to strum a note according to the expected level of loudness based on the music expressive intention. Finally, *metrical strength* was found to be informative for duration variation prediction which seems intuitive since the note's duration is often used to emphasize the metrical strength or weakness of notes in a melody.

### 4.2. Relationship with previous rule models

The duration and energy rules induced in this paper were compared with the rules obtained by Widmer (2003, 2002) (applying machine learning techniques to a data set of 13 performances of Mozart piano sonatas) as well as with the rules obtained by Friberg et al. (2006) (using an analysis by synthesis approach). Duration rule D3 is consistent with Widmer's TL2 rule “*Lengthen a note if it is followed by a substantially longer note*,” which may imply that the note in consideration is short. However, it contradicts its complementary condition TL2a (“*Lengthen a note if it is followed by a longer note and if it is in a metrically weak position*”). This might be due to the fact that note accentuation in jazz differ considerably from note accentuation in a classical music context, e.g., in case of swinging quavers, the first quaver (stronger metrical position) is usually lengthen. This however, is consistent with Friberg's *inégales* rule [“*Introduce long-short patterns for equal note values (swing)*”]. Duration rule D2 can be compared with Widmer's rule TS2 (“*Shorten a note in fast pieces if the duration ratio between previous note and current note is larger than 2:1, the current note is at most a sixteen note, and it is followed by a longer note”*). Similarly, duration rule D2 and D3 are consistent with Friberg's *Duration-contrast* (“*Shorten relatively short notes and lengthen relatively long notes*”), as dotted half notes can be considered relatively long notes, and eight notes can be considered as relatively short notes. The rules take as preconditions the duration of the note and the tempo of the piece. Energy rules E1 and E2 are consistent with Friberg's *high-loud* (“*Increase sound level in proportion to pitch height*”) and *phrase-arch* (*Create arch-like tempo and sound level changes over phrases*") rules, as notes in an ascending context might be played softer and vice-versa. However, energy rule E3 contradicts *phrase-arch* rule. Energy rule E2 shares the interval condition of the next note of Widmer's DL2 rule (“*Stress a note by playing it louder if it forms the apex of an up-down melodic contour and is preceded by an upward leap larger than a minor third*”). In addition, Widmer's rules for attenuating dynamics of notes (play softer) and our energy rules share the fact that the rule preconditions include intervals with respect to neighbor notes.

All in all there are similarities between the rules induced in this paper and the rules reported in the literature. However, at the same time, there are differences and even opposite findings, fact that is expected given the different data sets considered in the studies. While there seems to be similarities in expressive patterns in both classical and jazz music, clearly, both traditions have their own peculiarities and thus it is expected to find different/contradictory rules.

### 4.3. Model evaluation

Tables 7, 8 shows the accuracy of each performance action model trained with information of all features considered, and trained with selected features only. Accuracy is measured as the percentage of correctly classified instances. A statistical significance test (paired *t*-test with significance value of 0.05 and DoF of 99) against the baseline (i.e., majority class classifier) was performed for each model/feature-set (8 in total), using the approach by Bouckaert and Frank (2004) based on a repeated k-fold cross-validation scheme (i.e., using 10 runs of 10-fold cross validation). Significance level was corrected to 0.0125 for multiple comparisons with the *Bonferroni* correction (Benjamini and Hochberg, 1995). The significance results are shown in Tables 7, 8.

**Table 7:** **Accuracy of models trained with all extracted features (Mean ± Std Dev)**.

**Dataset**	**Baseline**	**Ripper**	***p*****-val**
Ornamentation	66.67 ± 0.50	68.86 ± 4.95	0.2426
Duration	50.46 ± 0.51	51.04 ± 5.80	0.7739
Onset	53.63 ± 0.52	60.53 ± 4.27	1.336e-4°
Energy	43.28 ± 0.50	52.48 ± 4.60	1.588e-6°

*°Statistically significant improvement*.

*(p < 0.0125) w.r.t baseline classifier*.

**Table 8:** **Accuracy of models trained with selected features (Mean ± Std Dev)**.

**Dataset**	**Baseline**	**Ripper**	***p*****-val**
Ornamentation	66.67 ± 0.50	70.12 ± 4.34	0.024857
Duration	50.46 ± 0.51	56.11 ± 5.66	0.001603°
Onset	53.63 ± 0.52	63.07 ± 3.90	2.91e-9°
Energy	43.28 ± 0.50	52.21 ± 4.62	6.3e-6°

*°Statistically significant improvement*.

*(p < 0.0125) w.r.t baseline classifier*.

The difference between the results obtained and the accuracy of a baseline classifier, i.e., a classifier guessing at random, indicates that the audio recordings contain sufficient information to distinguish among the different classes defined for the four performance actions studied, and that the machine learning method applied is capable of learning the performance patterns that distinguish these classes. It is worth noting that almost every model produced significantly better than random classification accuracies. This supports our statement about the feasibility of training classifiers for the data reported. However, note that this does not necessary imply that it is feasible to train classifiers for arbitrary recordings or performer.

The accuracy of all models except the energy variation model improved after performing feature selection. The improvement found with feature selection is marginal in most cases. However, this shows that it suffices to take into account a small subset of features (i.e., five or less features) in order to be able to predict with similar accuracy the performance actions investigated. The selected features contain indeed sufficient information to distinguish among the different classes defined for the four performance actions studied.

### 4.4. Rules specificity—generality

It has to be noted that the obtained expressive rules are specific to the studied guitarist and in particular to the considered recordings. Thus, the rules are by no means *guaranteed* general rules of expressive performance in jazz guitar. Nevertheless, the induced rules are of interest since Grant Green is a musician recognized for his expressive performance style of jazz guitar. In order to assess the degree of performer-specificity of the rules induced from the Grant Green's recordings we have, similarly to Widmer (2003), applied the induced rules to performances of the same pieces performed by two other professional jazz guitar players. The two guitarists recorded the pieces while playing along with prerecorded accompaniment backing tracks, similarly to the Grant Green recording setting. We processed the recordings following the same methodology explained in Section 2.2. In Table 9, we summarize the coverage of the rules measured in terms of the *true positive* (TP) and *false positive* (FP) rate, which is the proportion of correctly and incorrectly identified positives, respectively. As seen in the first two rows of the table, no significant degradation on the rule coverage was found for ornamentation prediction, which might be a good indicator for generality the ornamentation rules. However, rules for duration, energy, and onset show a higher level of degradation, which may indicate that these performance actions vary among Grant Green and the other two musicians. Nevertheless, in order to fully validate this results a much larger number of performances should be taken into consideration.

**Table 9:** **Model performance measured as true/false positives on train data (Grant Green) and test data (Musicians 1 and 2)**.

		**No. of rules**	**Grant Green**		**Musician 1**		**Musician 2**	
			**TP rate (%)**	**FP rate (%)**	**TP rate (%)**	**FP rate (%)**	**TP rate (%)**	**FP rate (%)**
Ornament	Yes	4	52.4	13.3	60.6	30.6	52.5	22.3
	No	(default)	86.7	47.6	69.4	39.4	77.7	47.5
Dur rat	Lengthen	1	50	9.4	32	12.7	27	16.5
	Shorten	2	51	2.4	45.2	5.2	31	8.4
Energy	Forte	2	34.7	11.7	17.1	18.5	24.4	18.9
	Piano	1	21.3	5.8	13.6	6.9	15.9	10.4
Onset dev	Advance	3	38.6	6.6	1.9	10.8	7.1	4.8
	Delay	1	49.8	10.4	28.8	36.5	29.9	25.2

## 5. Conclusions

In summary, we have presented a machine learning approach to obtain rule models for ornamentation, duration, onset, and energy expressive performance actions. We considered 16 polyphonic recordings of American jazz guitarist Grant Green and the associated music scores. Note, descriptors were extracted from the scores and audio recordings were processed in order to obtain a symbolic representation of the notes the main melody. Score to performance alignment was performed in order to obtain a correspondence between performed notes and score notes. From this alignment expressive performance actions were quantified. After discretizing the obtained performance actions we induced predictive models for each performance action prediction by applying a machine learning (sequential covering) rule learner algorithm. Extracted features were analyzed by applying (both filter and wrapper) feature selection techniques. Models were evaluated using a 10-fold cross validation and statistical significance was established using paired *t*-test with respect to a baseline classifier. Concretely, the obtained accuracies (over the base-line) for the ornamentation, duration, onset, and energy models of 70%(67%), 56%(50%), 63%(54%), and 52%(43%), respectively. Both the features selected and model rules showed musical significance. Similarities and differences among the obtained rules and the ones reported in the literature were discussed. Pattern similarities between classical and jazz music expressive rules were identified, as well as expected dissimilarities expected by the inherent particular musical aspects of each tradition. The induced rules specificity/generality was assessed by applying them to performances of the same pieces performed by two other professional jazz guitar players. Results show a consistency in the ornamentation patterns between Grant Green and the other two musicians, which may be interpreted as a good indicator for generality of the ornamentation rules.

## Author contributions

This work was developed as part of the Ph.D. research of SG, and under the supervision of RR. The tasks involved in this work are: 1. Data gathering; 2. Recording processing; 3. Data analysis; 4. Experiments designs; and 5. Reporting and writing.

### Conflict of interest statement

The authors declare that the research was conducted in the absence of any commercial or financial relationships that could be construed as a potential conflict of interest.
